# Identification and Validation of Protein Biomarkers of Response to Neoadjuvant Platinum Chemotherapy in Muscle Invasive Urothelial Carcinoma

**DOI:** 10.1371/journal.pone.0131245

**Published:** 2015-07-31

**Authors:** Alexander S. Baras, Nilay Gandhi, Enrico Munari, Sheila Faraj, Luciana Shultz, Luigi Marchionni, Mark Schoenberg, Noah Hahn, Mohammad Hoque, David Berman, Trinity J. Bivalacqua, George Netto

**Affiliations:** 1 Department of Pathology, Johns Hopkins School of Medicine, Baltimore, Maryland, United States of America; 2 Department of Urology, Johns Hopkins School of Medicine, Baltimore, Maryland, United States of America; 3 Department of Oncology, Johns Hopkins School of Medicine, Baltimore, Maryland, United States of America; 4 Department of Urology, Albert Einstein College of Medicine, Bronx, New York, United States of America; 5 Department of Otolaryngology, Johns Hopkins School of Medicine, Baltimore, Maryland, United States of America; 6 Department of Pathology, Queen's University, Kingston, Ontario, Canada; Biomedical Research Foundation, Academy of Athens, GREECE

## Abstract

**Background:**

The 5-year cancer specific survival (CSS) for patients with muscle invasive urothelial carcinoma of the bladder (MIBC) treated with cystectomy alone is approximately 50%. Platinum based neoadjuvant chemotherapy (NAC) plus cystectomy results in a marginal 5-10% increase in 5-year CSS in MIBC. Interestingly, responders to NAC (<ypT2) have a 5-year CSS of 90% which is in stark contrast to the 30-40% CSS for those whose MIBC is resistance to NAC. While the implementation of NAC for MIBC is increasing, it is still not widely utilized due to concerns related to delay of cystectomy, potential side-effects, and inability to predict effectiveness. Recently suggested molecular signatures of chemoresponsiveness, which could prove useful in this setting, would be of considerable utility but are yet to be translated into clinical practice.

**Methods:**

mRNA expression data from a prior report on a NAC-treated MIBC cohort were re-analyzed in conjunction with the antibody database of the Human Protein Atlas (HPA) to identify candidate protein based biomarkers detectable by immunohistochemistry (IHC). These candidate biomarkers were subsequently tested in tissue microarrays derived from an independent cohort of NAC naive MIBC biopsy specimens from whom the patients were treated with neoadjuvant gemcitabine cisplatin NAC and subsequent cystectomy. The clinical parameters that have been previously associated with NAC response were also examined in our cohort.

**Results:**

Our analyses of the available mRNA gene expression data in a discovery cohort (n = 33) and the HPA resulted in 8 candidate protein biomarkers. The combination of GDPD3 and SPRED1 resulted in a multivariate classification tree that was significantly associated with NAC response status (Goodman-Kruskal γ = 0.85 p<0.0001) in our independent NAC treated MIBC cohort. This model was independent of the clinical factors of age and clinical tumor stage, which have been previously associated with NAC response by our group. The combination of both these protein biomarkers detected by IHC in biopsy specimens along with the relevant clinical parameters resulted in a prediction model able to significantly stratify the likelihood of NAC resistance in our cohort (n = 37) into two well separated halves: low-26% n = 19 and high-89% n = 18, Fisher’s exact p = 0.0002).

**Conclusion:**

We illustrate the feasibility of translating a gene expression signature of NAC response from a discovery cohort into immunohistochemical markers readily applicable to MIBC biopsy specimens in our independent cohort. The results from this study are being characterized in additional validation cohorts. Additionally, we anticipate that emerging somatic mutations in MIBC will also be important for NAC response prediction. The relationship of the findings in this study to the current understanding of variant histologic subtypes of MIBC along with the evolving molecular subtypes of MIBC as it relates to NAC response remains to be fully characterized.

## Introduction

Muscle invasive urothelial carcinoma of the bladder (MIBC) has traditionally been treated by radical cystectomy and pelvic lymphadenectomy[1,2]. Recent evidence from randomized controlled trials[3] and a meta-analysis thereof[4] have supported a marginal benefit for platinum-based combination neoadjuvant chemotherapy (NAC) prior to cystectomy, in improving survival when examining all-comers. NAC utilization in MIBC is increasing with the National Cancer Database reported increasing utilization of NAC, from 10.2% in 2006 to 20.9% in 2010.[5] Additionally, almost 80% of the Bladder Cancer Advocacy Network (BCAN) oncologists offer NAC with gemcitabine/cisplatin (GC) the most utilized regimen (90%), followed by methotrexate/vinblastine/adriamycin/cisplatin (MVAC) (30%) and dose-dense (DD)-MVAC (20%).[6]

The barriers to more uniform implementation of NAC for MIBC include concerns for delay of surgery and risk of disease progression along with the apparent modest survival benefit of NAC, absolute magnitude of ~ 5–10%[4]. Careful review of the randomized controlled trials of platinum-based combination NAC[3] highlights an important phenomenon: patients who achieve a pathologic response to NAC have a 5 year survival rate of approximately 80–90% while those with NAC resistant (NR) MIBC have a 5 year survival rate of approximately 30–40%, which is a robust difference and notably different than the 50% 5-year survival for patients with MIBC treated by cystectomy alone. We hypothesize that while only a modest 5–10% benefit in 5 year survival is observed with NAC in all-comers, appropriate patient stratification based on the likelihood that a patient will benefit from NAC should yield clinically actionable data.

Such a stratification scheme would require improvements in our ability to (*i*) identify MIBC patients that harbor occult micro/macro metastases and (*ii*) identify MIBC patients whose tumors are responsive to a NAC regiment. If such stratification were possible and only patients with a high likelihood of benefiting from NAC were treated, then we expect that the absolute magnitude of the benefit from NAC (in terms of post-cystectomy overall survival) will become significantly larger, as suggested by the literature highlighted above [3]. Recent investigations have identified molecular signatures of these two concepts primarily via microarray based high-throughput gene expression profiling technologies [7–10], including a prior study by an author of this study that identified a molecular signature able to identify MIBC likely to exhibit lymph node metastases at cystectomy [11].

Despite compelling evidence supporting the hypothesis that a molecular signature can identify patients likely to benefit from NAC, these results have not yet translated into clinically useful tools to better guide the management of patients with bladder cancer. Ultimately, generating clinically useful tools from the findings of these prior investigations [7–11], will require that the molecular signature(s) can be detected with established methodologies utilized in the modern anatomic and molecular pathology laboratories from routine formalin fixed paraffin embedded specimens. Additionally, these predictive models will need to combine the relevant clinical, pathologic, and molecular features in order to maximize their applicability and accuracy. In this report we will limit our study to clinical and molecular factors predictive of response to GC NAC in MIBC from the therapy naive tissue biopsy material.

## Results

### Patient demographics and clinical parameters associated with response to neoadjuvant chemotherapy

417 patients underwent RC for MIBC between 2000 and 2013 at our institution. The NAC utilization rate was approximately 50%. In cases where GC NAC was administered, clinical follow-up, pathology, and NAC dosing data was available for 176 patients, comprising our GC NAC cohort. The chemo naive tissue biopsy materials were available for 37 patients (GC NAC TMA cohort) and were incorporated into tissue microarrays for further analysis. A cohort of 121 consecutive patients treated by cystectomy alone between 2000 and 2005 with complete clinical and pathologic data was used for comparison (no NAC cohort). No significant differences were identified when comparing the GC NAC TMA cohort to the remainder of the GC NAC cohort or when comparing the whole GC NAC cohort to the no NAC cohort regarding pre-operative clinical parameters, Table 1.

**Table 1:** Muscle invasive urothelial carcinoma of the bladder cohorts.

	GC NAC		*p-value* ^*a*^	no NAC	*p-value* ^*b*^
	(no TMA)	(TMA)			
Number of patients	139	37		121	
Median Age (range)	62 (41–82)	63 (44–83)	*0*.*3832*	64 (34–88)	*0*.*2090*
Gender					
Male	79%	84%	*0*.*6463*	82%	*0*.*7656*
Female	21%	16%		18%	
Race					
Caucasian	94%	86%	*0*.*1756*	90%	*0*.*6767*
Non-Caucasian	6%	14%		10%	
Clinical Stage					
cT2	72%	60%	*0*.*2802*	74%	*0*.*4658*
cT3	22%	35%		21%	
cT4	7%	5%		5%	

Patient preoperative clinical demographics based on cohort group. No statistically significant differences were observed within the GC NAC cohort based on the availability of tissue for TMA incorporation (^a^) or across the NAC and no NAC cohorts (^b^). Medians were compared by Kruskal-Wallis tests, all others were Fisher’s exact tests.

### Identification of a putative mRNA signature of response to neoadjuvant chemotherapy MIBC

**Fig 1:**
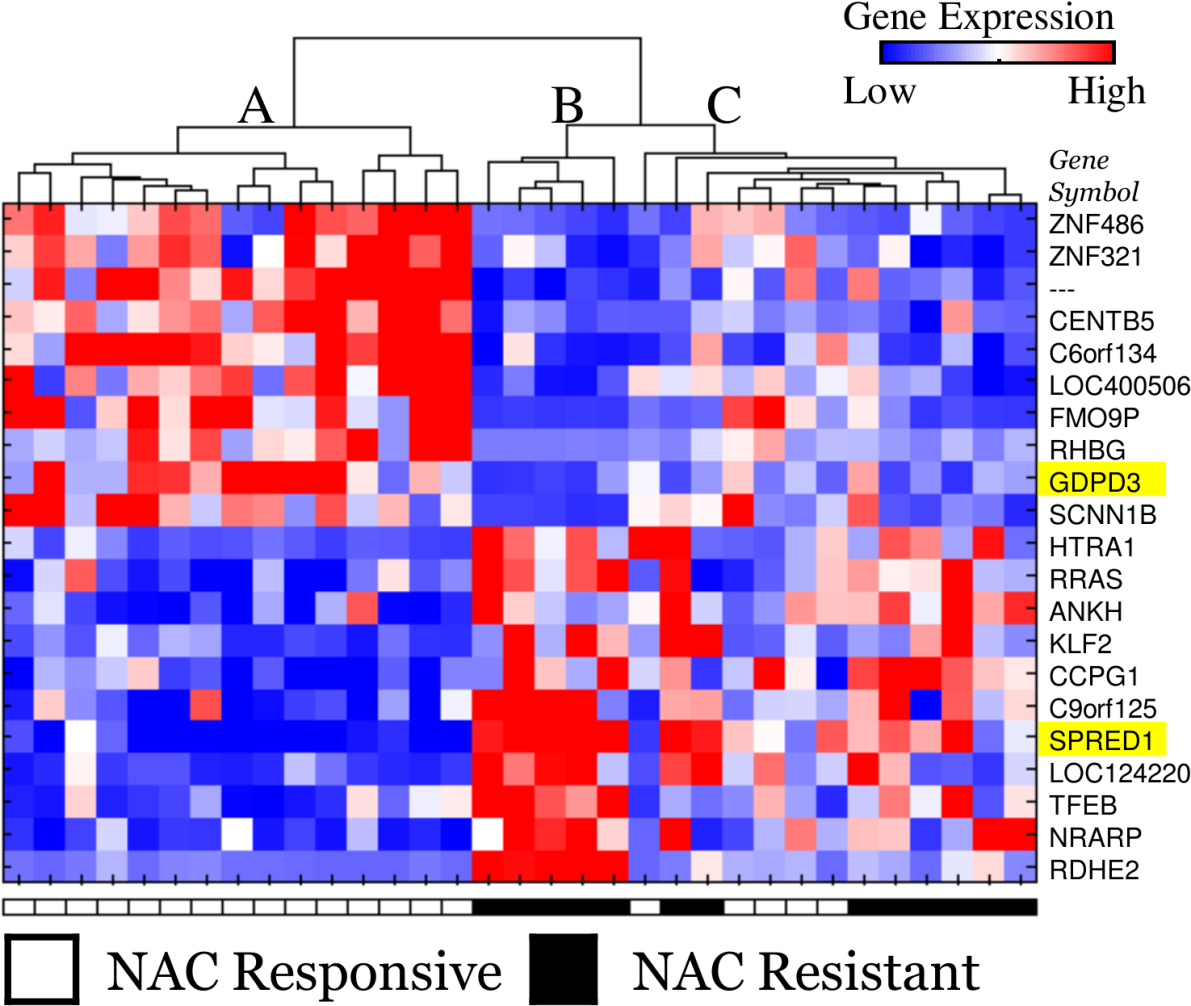
Gene expression patterns of response and resistance to NAC in MIBC. The heatmap and hierarchical dendrogram of the samples (rows—mRNA, columns—samples) using the 21 target mRNA species identified from the Kato et al. cohort shows robust differences that correlate well to NAC response status. Unsupervised clustering revealed three distinct yet related sample groups designated as A, B, and C in the dendrogram. The two genes selected in the IHC based prediction model are highlighted in yellow.

We examined mRNA expression profiling data from a previous study by Kato et al[7] of NAC naive biopsy materials from MIBC in the context of combination gemcitabine/platinum NAC. The distribution of NAC response in these data exhibits a clear bi-modal distribution, dividing the patients into NAC responsive (R) and NAC resistant (NR) based on reduction of tumor volume by radiographic assessment, as described in that report. Our analysis of these data resulted in a set of 21 target mRNAs in which the groupwise p-value is less than 0.005 and the false discovery rate for these 21 targets is <10%. The analysis originally reported on of these data identified 14 mRNAs, which exhibited a median univariate AUC of 0.734 with respect to responder status; in contrast, the 21 mRNA identified by our analyses, described in the methods section, resulted in a statistically increased median AUC of 0.845 (Mann-Whitney p-value 0.001). Unsupervised clustering yielded three distinct yet related groups as depicted in Fig 1; with group A corresponding to the molecular signature of NAC responsiveness, group B corresponding to the molecular signature of NAC resistance, and group C corresponding to an indeterminate response status. Interestingly, the molecular signature of group A is effectively the opposite of group B and Group C depicts an intermediary between these two patterns, which correlates to the rate of NAC response observed in these groups as depicted in Fig 1.

### Translation of the mRNA signature into an immunohistochemical panel predictive of GC NAC response

The 21 mRNA targets identified from the gene expression profiling data were then characterized via the Human Protein Atlas (HPA), http://www.proteinatlas.org [12]. Briefly, the HPA is a repository of scanned tissue microarrays (TMAs) of normal and cancer tissues that have been stained by immunohistochemistry (IHC) for over 24,000 commercially available antibodies representing nearly 17,000 unique proteins. Importantly, since the HPA includes small cohorts of each cancer type characterized, one can assess for the presence of at least some degree of differential staining across urothelial carcinomas. 8 of the 21 mRNA targets had commercially available antibodies to detect their respective protein products via IHC and exhibited at least 10% differential staining across the cohort of urothelial carcinomas present in the HPA.

**Fig 2:**
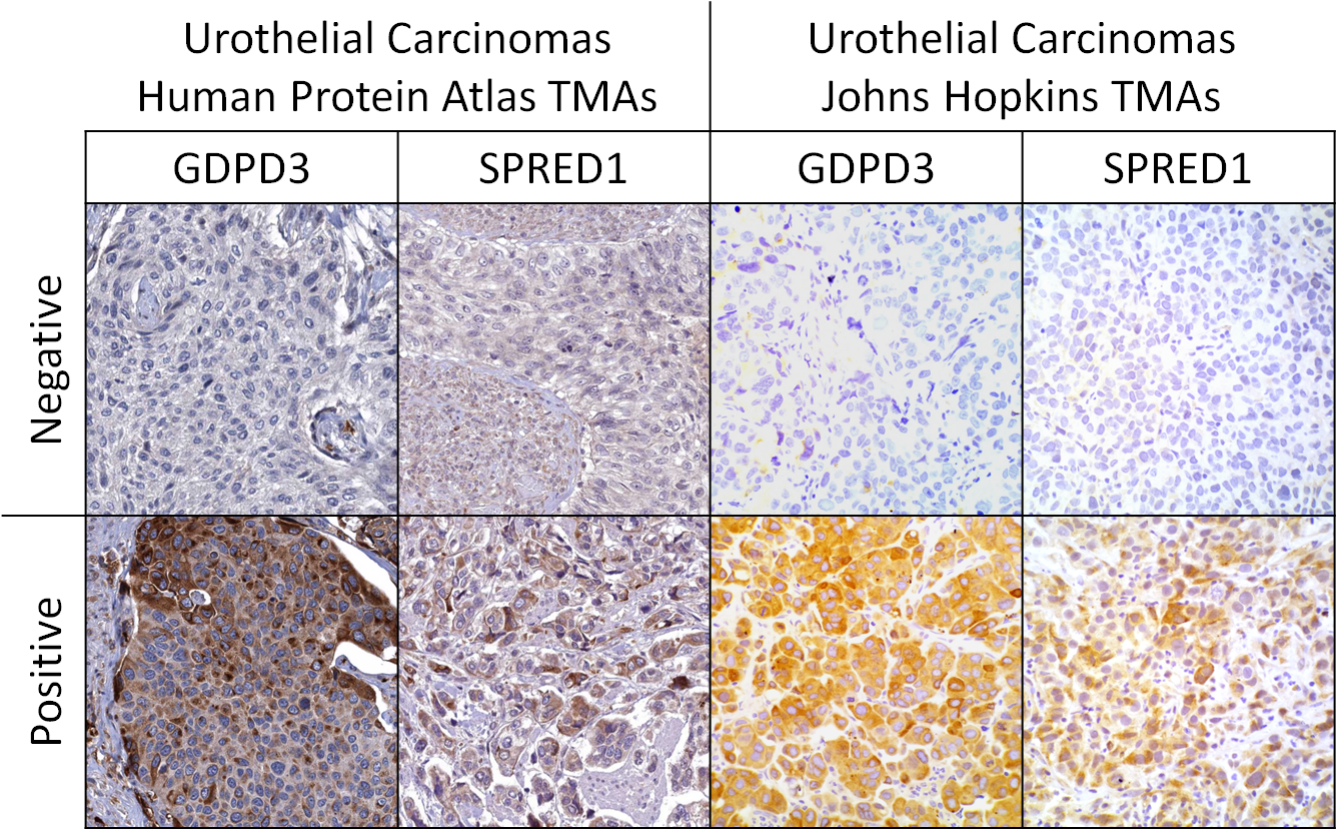
Representative IHC for GDPD3 and SPRED1 from the HPA and our MIBC cohort. The staining patterns in the cohort of urothelial carcinomas present within the Human Protein Atlas with the same antibodies used in this study, are recapitulated in our cohort of muscle invasive urothelial carcinoma of the bladder.

We were able to validate 6 of these 8 antibodies, as described in the methods section, which were then applied to TMAs of therapy naive biopsy materials of muscle invasive urothelial carcinoma from our institution who were treated with GC NAC (n = 37). In addition to these 6 antibodies, we also characterized our TMA MIBC cohort with respect to Ki67 staining, a semi-quantitative index of proliferation, and p53 mutation status, as detected by IHC[13]. We limited the statistical modeling of NAC resistance to a maximum of two IHC markers for both practical reasons with respect to the utilization of IHC in the work-up of a case by a pathologist and statistical considerations given the relatively smaller sized data set of our TMAs and the number of independent predictors. We employed adaptive boosting[14] in conjunction with multivariate classification tree to develop a classification tree capable of stratifying the likelihood of NAC resistance. The combination of the expression status of GDPD3 and SPRED1 by IHC (Fig 2) was selected as the best pair of markers based on adaptive boosting, S1 Table. The Cohen’s kappa statistics for these two markers as scored by two independent pathologists were each very high (> 0.9).

**Fig 3:**
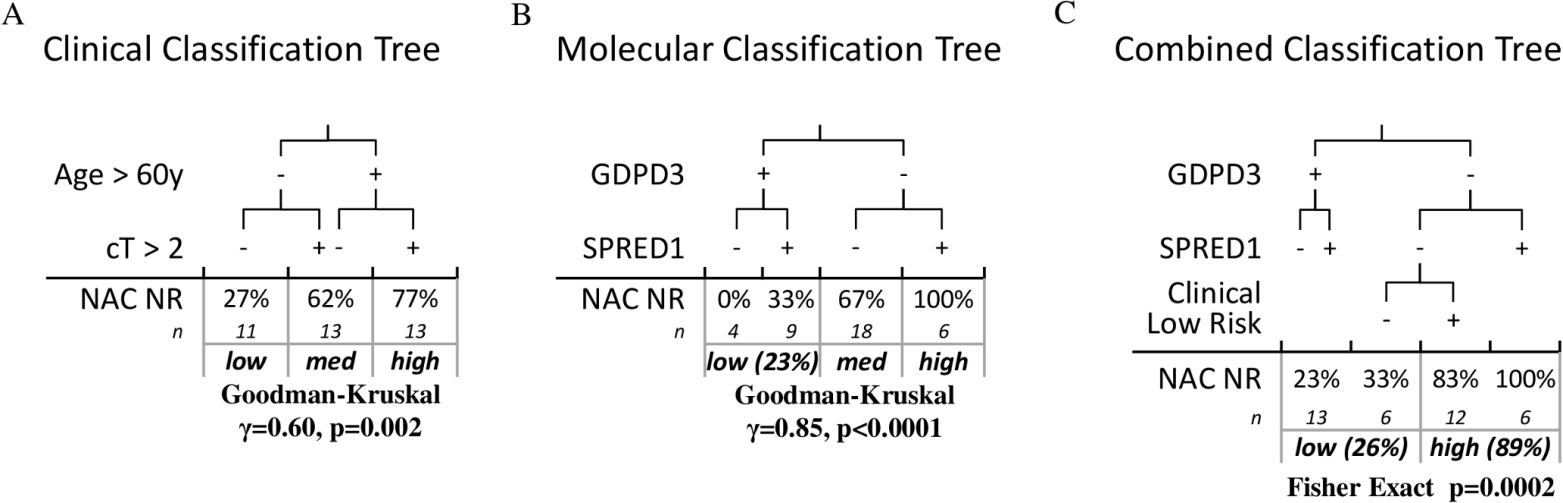
(A) The application of a previously developed classification tree based on the clinical parameters of age greater than 60 and clinical stage greater than cT2 is significantly associated with NR rate in the GC NAC TMA cohort. (B) A multivariate classification tree based on the IHC staining of GDPD3 and SPRED1 is also significantly associated with NR rate in the GC NAC TMA cohort. (C) A multivariate classification tree combining the IHC staining of GDPD3 and SPRED1 along with the relevant clinical factors (clinical low risk = age≤60 & cT≤2) simplifies the stratification of NAC resistance into two well separated halves.

The stability of the selection of the above proteins was 99% over 100x 10-fold cross-validation. As shown in Fig 3B, positive GDPD3 protein expression is associated with response to GC NAC while positive SPRED1 protein expression is associated GC NAC resistance. Importantly, these associations observed at the protein level in our independent MIBC NAC cohort are the same as was observed in the discovery cohort[7] at the mRNA level, highlighted in yellow in Fig 1.

Given that response to NAC is strongly associated with post cystectomy CSS, it is formally possible that the IHC markers we have identified to correlate with response to NAC may be just be prognostic of post-cystectomy CSS as opposed to truly predictive of NAC response. Since the mRNA and protein changes were concordantly associated to response, we examined GDPD3 and SPRED1 gene expression data in three publically available data sets of non-NAC treated MIBC (GSE48075[4], GSE32894[5], and GSE31684[15]). A significant (p<0.05) association of the expression of GDPD3 or SPRED1 to cancer specific survival from these data was not identified by Cox proportional hazard modeling across these three datasets. These findings support the notion that GDPD3 and SPRED1 are markers predictive of NAC response and not just prognostic of post cystectomy CSS.

### Combination of clinical and IHC protein based models of GC NAC response

From our recent report of this GC NAC cohort[16], univariate and multivariate analyses revealed that age > 60 years at cystectomy and clinical stage > cT2 were independent factors significantly associated with NAC resistance (NR), p<0.05. Sex, race, and clinical LN status were not significantly associated with GC NAC response status. We applied the classification tree we previously developed from these clinical parameters[16] to the 37 patients who biopsy materials were incorporating into TMAs. Briefly, this classification tree stratifies patients into three tiers of likelihood of NAC resistance: low ~25%, intermediate ~50%, and high ~75%. The application of the classification tree to the GC NAC TMA cohort resulted in a similar statistically significant stratification of the likelihood of NAC resistance: low 27%, intermediate 62%, high 77% (Goodman-Kruskal γ = 0.60 p = 0.002) Fig 3.

These two distinct models, one derived from clinical factors and the other from protein markers assayed by IHC in these tissues, are both strongly statistically associated with NAC resistance, Fig 3A and 3B. Additionally, these two models are independent of one another (Goodman-Kruskal γ = 0.09 p = 0.673) and are independently associated to NAC resistance on multivariate analysis (multivariate logistic regression p<0.05). A combined multivariate classification tree, incorporating both of these models together, resulted in a prediction model able to significantly stratify the likelihood of NAC resistance in our cohort (n = 37) into two well separated halves: low-26% n = 19 and high-89% n = 18 (Fisher’s exact test, p = 0.0002).

## Materials and Methods

### Clinical cohort

The study was approved by the Johns Hopkins Institutional Review Board. Informed consent was not required because after the data were collected they were analyzed anonymously. We queried the Johns Hopkins Hospital (JHH) Institutional Review Board approved Bladder cancer database to identify patients who received any NAC followed by open RC between 2000 and 2013. Consecutive MIBC patients treated by RC alone (no NAC cohort) beginning in the year 2000 were identified and used for comparison. These patients were either not offered NAC as it was not currently standard of care at our institution during that time period, or chose not to pursue NAC. Patients with unknown follow-up or cause of death were excluded.

### Neoadjuvant Chemotherapy Regimens

All patients were treated with gemcitabine and cisplatin (GC) NAC regimens for greater than 2 cycles. GC regimens assessed included (1) the traditional gemcitabine 1000mg/m^2^ on days 1, 8, and 15 along with cisplatin 70mg/m^2^ on day 1 of a 28-day cycle for 3–4 cycles, (2) gemcitabine 1000mg/m^2^ on days 1 and 8 along with cisplatin 70mg/m^2^ on day 1 of a 21-day cycle for 4 cycles, or (3) gemcitabine 1000mg/m^2^ and cisplatin 35mg/m^2^ given on days 1 and 8.

### Disease Assessments

Each NAC patient received a pre-chemotherapy staging CT or MRI. Following NAC, a restaging examination was performed within one month prior to RC, comprised of diagnostic cystoscopy without transurethral resection (TUR) and CT or MRI of chest, abdomen, and pelvis. NAC patients with clinical node positive disease (pre- or post-chemotherapy) were included if their disease was deemed surgically resectable or lymph node (LN) enlargement was confined to the pelvis. All patients underwent pre-operative imaging and all non-NAC patients with LN disease/metastasis were excluded and recommended to receive systemic chemotherapy unless deemed surgically resectable. Pelvic lymphadenectomy followed a standard surgical template including LN of the obturator fossa and those along the internal and external iliac arteries up to and including the common iliac artery and vein.

### Pathologic Assessments

All pathologic evaluations were performed at JHH by expert pathologists. Included patients were required to have histologically confirmed urothelial carcinoma of the bladder diagnosed on pre-operative transurethral resection of bladder tumor (TURBT) biopsy. Patients with variant histologies were included provided the majority of the lesion exhibited conventional urothelial morphology. Patients with any small cell histology were excluded. NAC responders (R) were defined by the absence of residual MIBC (<ypT2) at cystectomy; conversely NAC resistant (NR) tumors were defined by the presence of residual muscle-invasive (≥ypT2) at cystectomy. CSS was defined according to review of death certificates by the JHH Cancer Registry or biopsy of metastatic lesions confirming UC and updated for all patients by review of clinical medical records and query of the Social Security Death Database. For cases without evidence of cancer-specific death, survival was censored at the date of last clinic visit.

### Tissue microarray construction and immunohistochemistry

Tissue microarrays were constructed at the Johns Hopkins tissue microarray facility utilizing 1.0 mm cores in triplicate from the same sample, when possible. Antibodies were acquired from commercial sources as follows: GDPD3 Sigma-HPA041470, ZNF816 Sigma-HPA051271, CCPG1 Sigma-HPA026861, SCNN1B Sigma-HPA015612, SPRED1 Sigma-HPA042193, RDHE2 Sigma-HPA025224. Antigen retrieval was with a citrate 25 min steam for all antibodies. These antibodies were utilized at the following dilutions for staining of sections of formalin fixed paraffin embedded tissue for a 45 incubation at room temperature: GDPD3 1:100, ZNF816 1:500, CCPG1 1:200, SCNN1B 1:100, SPRED1 1:100, RDHE2 1:200. Detection of immunolabeling was performed using anti-mouse or anti-rabbit HRP conjugated secondary antibodies and counterstaining was performed with DAB. The expected pattern of staining in normal tissues for these reagents as characterized in the human protein atlas (www.proteinatlas.org [12]) were reproduced in a panel of normal tissues included in the TMAs of this study in order to validate the antibodies expected staining.

These 6 antibodies were scored in blinded manner with respect to NAC response status by two practicing pathologists (ASB & GN) and reported in binary format as positive or negative based on the predefined criteria of greater than 10% of at least moderate staining (2 out of a the 0–3 scale) assessed over the available cores for a given sample present in the tissue microarrays examined.

### Bioinformatic and statistical analyses

With the goal of translating gene expression data to the protein/IHC level, we filtered for large mRNA expression changes with mean signal < 300 in one group and subsequently mean signal > 500 in the second group (Affymetrix MAS 5.0 arbitrary units). This simple filter reduced the possible targets from ~ 54000 to ~ 300, which also helps to avoid overfitting in the subsequent statistical analyses. Next, we utilized area under the receiver operator curve analysis to characterize which of these robust mRNAs also exhibit favorable statistical characteristics. These results were then correct for multiple testing via random permutation analysis (200x) to arrive at the 21 target mRNAs.

Fisher exact test, Goodman-Kruskal test, and Cohen’s Kappa statistic along with multivariate logistic regression and multivariate classification tree modeling were performed using the MATLAB (version 8.4). The immunohistochemical markers were selected by adaptive boosting. Briefly, in the first round of selection all samples were weighted equally, reducing to univariate tests of association. In the second round of selection, the samples were weighted by the errors of the first marker (i.e. predictor variable) selected. The stability of the marker selection process was accessed using 100x 10-fold internal cross-validation. All analyses were performed using the commercial software package MATLAB 8.4 (The MathWorks Inc., Natick, MA, 2000).

## Discussion

In this study we present a logical approach to the development of a predictive model of resistance to neoadjuvant gemcitabine/cisplatin chemotherapy of muscle invasive urothelial carcinoma; starting with a discovery gene expression data set, combining with databases of antibodies for IHC, and then testing on an independent cohort of MIBC from our institution. Importantly, the two antibodies utilized to detect these proteins were readily utilized on routine FFPE pathologic specimens, highlighting the feasibility of the overall approach. Additionally, the associations that the respective mRNA species exhibited to GC NAC in the gene expression profiling discovery cohort from the literature[7] were maintained in the independent MIBC cohort from our institution at the protein level as presented in this study.

Not entirely unexpectedly, the combination of both clinical and molecular factors yielded the most promising results, as shown in Fig 3, ultimately resulting in a simplified two tiered stratification of the likelihood of NAC resistance dividing the GC NAC TMA cohort into two halves: low risk (23%) and high risk (89%). The identification of a patient stratum with a putative NAC resistance rate of almost 90%, representing half of the cohort, coupled with the poor post-cystectomy cancer specific survival of patients whose MIBC proves to be resistant to NAC at cystectomy (30%) as compared to MIBC patients treated by cystectomy alone (~50%), highlights that if appropriately validated such a predictive model could be particularly relevant to the management of these types of MIBC patients. Alterative therapeutic modalities that could be considered to GC NAC would include DD-MVAC, other NAC, or directly to cystectomy as to avoid a delay to cystectomy without any other intervention likely to benefit the patient[17,18]. This naturally then raises the question of whether the factors identified in this report to be predictive of response to GC NAC would translate to similar platinum based NAC or not. We focused this effort on GC NAC given that is was the more common NAC regimen utilized at our institution, and would thereby allow us to achieve adequate numbers in a timely manner; however, we anticipate that as we test external validation cohorts, many will contain both GC NAC and other NAC (such as MVAC), which will thereby allow us to test this hypothesis.

The results from our study are limited by sample size and the fact that it represents the findings from only our institution. Additional validations across multiple institutions are warranted and are currently being drafted to refine the predictive models developed in this report. In this study we have focused primarily on proteins, and indirectly mRNA. However, with the completion of the TCGA sequencing efforts in MIBC[19] and a subsequent exome level sequencing in a NAC MIBC cohort[20], we anticipate that somatic mutations (such as ERCC2, ATM, RB1, FANCC, and others) in urothelial carcinoma will likely also play a role in identifying patients likely to derive benefit from platinum based NAC in MIBC[21,22]. Furthermore, the relationship of these molecular factors to the current understanding of variant histologic subtypes[23] along with evolving molecular subtypes of urothelial carcinoma[24–29] as it relates to responsiveness to platinum based chemotherapy remains to be fully characterized.

## Supporting Information

S1 Table Adaptive boosting applied to the NAC cohort identifying the best pair of IHC markers predictive of response in muscle invasive urothelial carcinoma. (XLSX)

S2 Table De-identified cohort data including the clinical and molecular factors analyzed in this study. (XLSX)
