# Correction: Identification and Validation of Protein Biomarkers of Response to Neoadjuvant Platinum Chemotherapy in Muscle Invasive Urothelial Carcinoma

**DOI:** 10.1371/journal.pone.0143990

**Published:** 2015-11-24

**Authors:** Alexander S. Baras, Nilay Gandhi, Enrico Munari, Sheila Faraj, Luciana Shultz, Luigi Marchionni, Mark Schoenberg, Noah Hahn, Mohammad Obaidul Hoque, David Berman, Trinity J. Bivalacqua, George Netto

The ninth author’s name appears incorrectly. The correct name is: Mohammad Obaidul Hoque.

Fig 3 is incorrect. Please view the corrected Fig 3 here.

**Fig 3:**
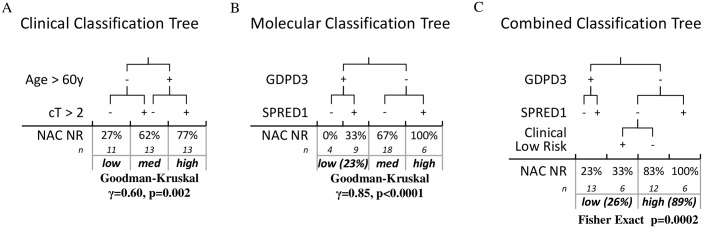
(A) The application of a previously developed classification tree based on the clinical parameters of age greater than 60 and clinical stage greater than cT2 is significantly associated with NR rate in the GC NAC TMA cohort. (B) A multivariate classification tree based on the IHC staining of GDPD3 and SPRED1 is also significantly associated with NR rate in the GC NAC TMA cohort. (C) A multivariate classification tree combining the IHC staining of GDPD3 and SPRED1 along with the relevant clinical factors (clinical low risk = age≤60 & cT≤2) simplifies the stratification of NAC resistance into two well separated halves.
